# Phage-antibiotic combinations in various treatment modalities to manage MRSA infections

**DOI:** 10.3389/fphar.2024.1356179

**Published:** 2024-04-09

**Authors:** Archana Loganathan, Bulent Bozdogan, Prasanth Manohar, Ramesh Nachimuthu

**Affiliations:** ^1^ School of Bioscience and Technology, Vellore Institute of Technology (VIT), Vellore, India; ^2^ Medical Microbiology Department, Adnan Menderes University, Aydin, Türkiye

**Keywords:** phage-antibiotic synergy, combination therapy, *Staphylococcus aureus*, *Galleria mellonella*, treatment strategy

## Abstract

**Introduction:** The emergence of antibiotic resistance is a significant challenge in the treatment of bacterial infections, particularly in patients in the intensive care unit (ICU). Phage-antibiotic combination therapy is now being utilized as a preferred therapeutic option for infections that are multi-drug resistant in nature.

**Methods:** In this study, we examined the combined impact of the staph phage vB_Sau_S90 and four antibiotics on methicillin-resistant *Staphylococcus aureus* (MRSA). We conducted experiments on three different treatment sequences: a) administering phages before antibiotics, b) administering phages and antibiotics simultaneously, and c) administering antibiotics before phages.

**Results:** When the media was supplemented with sub-inhibitory concentrations of 0.25 μg/mL and 1 μg/mL, the size of the plaque increased from 0.5 ± 0.1 mm (in the control group with only the phage) to 4 ± 0.2 mm, 1.6 ± 0.1 mm, and 1.6 ± 0.4 mm when fosfomycin, ciprofloxacin, and oxacillin were added, respectively. The checkerboard analysis revealed a synergistic effect between the phages and antibiotics investigated, as indicated by a FIC value of less than 0.5. The combination treatment of phages and antibiotics demonstrated universal efficacy across all treatments. Nevertheless, the optimal effectiveness was demonstrated when the antibiotics were delivered subsequent to the phages. Utilizing the *Galleria mellonella* model, *in vivo* experiments showed that the combination of phage-oxacillin effectively eliminated biofilm-infected larvae, resulting in a survival rate of up to 80% in the treated groups.

**Discussion:** Our findings highlight the advantages of using a combination of phage and antibiotic over using phages alone in the treatment of MRSA infections.

## Introduction

Bacterial antibiotic resistance arises from alterations or adaptations to the medications employed for treatment, and in some cases, it can also stem from inherent resistance (Murray et al., 2022). The diminishing effectiveness of antibiotics in treating drug-resistant bacteria has led to the resurgence of phage therapy as a contemporary medical approach. Phage therapy utilizes bacteriophages, which are bacterial viruses, to address bacterial illnesses that are resistant to antibiotics (Lin et al., 2017). A 2016 antimicrobial assessment projected that the annual death toll from antimicrobial resistance (AMR) would rise to 10 million by 2050 (O’Neill, 2016). *Staphylococcus aureus* is a well-known pathogen that has a tendency to quickly acquire resistance. The confirmed evolution of antibiotic resistance in *Staphylococcus aureus* has established it as the primary infection responsible for over 100,000 fatalities in 2019 (Murray et al., 2022). These examples indicate that *S. aureus* has evolved into a lethal pathogen that demands urgent attention and a different therapeutic approach.

In recent years, phage therapy has gained prominence as a treatment option in some affluent nations (Pires et al., 2020). The majority of clinical findings about phage treatment have demonstrated encouraging therapeutic results (Mitropoulou et al., 2022). Furthermore, clinical case studies have shown promising outcomes in the treatment of osteomyelitis (Onsea et al., 2021), infections in the bones and joints (Aslam et al., 2020; Gibb and Hadjiargyrou, 2021), and respiratory infections (Mitropoulou et al., 2022). Several case studies have consistently found that the combination of phages with antibiotics yields superior results compared to phage treatment alone (Save et al., 2022a). Phage and antibiotic combination treatment refers to the utilization of phage and antibiotics at lower doses that function together, resulting in a combined effectiveness that surpasses the separate effects. The utilization of both phages and antibiotics might have clinical significance due to certain therapeutic advantages that surpass treatment with phages alone. For instance, when antibiotics and phages are used together at a concentration that does not completely suppress bacterial growth, it has been seen that the number of phages produced during infection increases (Ryan et al., 2012). Additionally, this combination has been shown to eliminate biofilms (Dickey and Perrot, 2019) and facilitate a trade-off in the evolution of bacteria (Chan et al., 2018). In future, this therapy can be selected for treatment based on the confirmed synergistic interaction between the antibiotic and phage. It is important to note that while certain antibiotics may have synergistic effects with a specific phage, they may not have the same impact when combined with other phages, leading to an antagonistic combination. For example, the drug rifampicin has an antagonistic effect when used together with staphylococcal phage sb-1 (Wang et al., 2020a). However, it has a synergistic effect when combined with phage SAP-26 (Rahman et al., 2011). The use of phage therapy to combat *S. aureus* has been extensively described previously (Capparelli et al., 2007; Pincus et al., 2015; Petrovic Fabijan et al., 2020; Plumet et al., 2022). The often-reported antibiotics utilized in conjunction with staphylococcal phages consist of penicillin (Himmelweit, 1945), gentamycin (Kirby, 2012), rifampicin (Rahman et al., 2011), linezolid (Chhibber et al., 2013), ciprofloxacin (Roszak et al., 2022), and vancomycin (Save et al., 2022b). Extensive study has been conducted on the topic of phage-antibiotic synergy (PAS) in *S. aureus* (Diallo and Dublanchet, 2022). However, there is a scarcity of publications focusing on the treatment sequence in PAS. The PAS methodology described in this paper is expected to effectively evaluate the efficacy of phage-antibiotic combination therapy in eradicating pathogens.

Recently, non-mammalian animal models, like *Galleria mellonella* and *Caenorhabditis elegans*, have become appealing options for studying bacterial infections and phage therapy in living organisms. *Galleria mellonella*, commonly referred to as the wax moth larva, is extensively employed as a model organism for investigating bacterial pathogenicity. *Galleria mellonella* has innate immune systems that exhibit numerous resemblances to those seen in mammals, including as the synthesis of antimicrobial peptides and the stimulation of phagocytic cells (Menard et al., 2021). Furthermore, *G. mellonella* is an excellent model for efficiently testing prospective antimicrobial drugs, such as phages, because of its compact size, brief lifespan, and convenient manageability (Jorjao et al., 2018). Prior research has shown that phage therapy is effective in treating *S. aureus* infections in *G. mellonella* larvae (Jeon and Yong, 2019; Wang et al., 2020b). Simple animal models are useful for conducting *in vivo* investigations to get significant insights into the safety and efficacy of phage therapy.

*In vitro* testing of phage vB_Sau_S90 showed it was active against *S. aureus* clinical isolates (Loganathan and Nachimuthu, 2022). The primary objective of the present study was to assess the efficacy of the phage when used in combination with four antibiotics (fosfomycin, ciprofloxacin, oxacillin, and vancomycin). We conducted an examination of the synergistic effects of various combinations of phages and antibiotics by the implementation of checkerboard analysis. The study investigated the phage-antibiotic combination utilizing three distinct treatment sequences: a) administering phages before antibiotics (PRE), b) administering phages and antibiotics simultaneously (SIM), and c) administering antibiotics before phages (POS). Ultimately, we investigated the efficacy of combined therapy of phages and oxacillin in enhancing the survival of infected larvae, *G. mellonella*.

## Materials and methods

### Bacteria, bacteriophage, and culture conditions

The clinical strains of *S. aureus*, namely, SA-28, SA-90, and SA-165, used in this study were obtained from a diagnostic laboratory located in Chennai, India. The isolates were derived from samples of pus and exhibited the presence of the *mec*A gene, so categorizing it as methicillin-resistant *S. aureus* (MRSA). For the *in vitro* experiments, only one isolate, SA-90, was employed. However, for the *in vivo* studies, all three isolates were used, taking into consideration the characteristics of their biofilms. Bacterial cultures were preserved in Brain Heart Infusion broth (Hi-Media, India) with the addition of agar when necessary. For biofilm studies, Tryptic Soy Broth (TSB) supplemented with 1% glucose was used (Hi-Media, India). The culture was incubated at 37°C.

This work utilized the previously identified staph phages, vB_Sau_S90 and vB_Sau_S165 (Loganathan and Nachimuthu, 2022). Both phages possess the capacity to eradicate all three bacterial strains used in this study. *In vitro* tests were conducted using the phage vB_Sau_S90 against SA-90. *In vivo* investigations, on the other hand, used both vB_Sau_S90 and vB_Sau_S165. The plaque assay was conducted using a 0.45% soft agar medium, and the phages were cultured and preserved according to a previously published method (Loganathan and Nachimuthu, 2022).

### Phage-antibiotic combination test

A synergy test was conducted using the bacterial strain SA-90 and phage vB_Sau_S90 according to the procedure described by Comeau et al., 2007 (Comeau et al., 2007). Briefly, a double agar overlay (DAOL) was conducted with the minimum concentration of phages, aiming to generate distinct and quantifiable plaques. Antibiotic discs were positioned on a desiccated overlay plate. The antibiotic disc comprises fosfomycin (200 μg), ciprofloxacin (5 μg), oxacillin (30 μg), and vancomycin (30 μg) (Hi-Media, India). The plates were placed in an incubator at 37°C for 16 h. The area where bacterial growth was inhibited and any alterations in the size of the plaques were documented. It is important to understand that our definition for sub-inhibitory concentration in the disc diffusion-DAOL test is based on the least antibiotic concentration in the gradient diffusion that did not affect the bacterial growth, thus enhancing the visible plaque formation (the region next to the zone of inhibition is considered a sub-lethal antibiotic dose).

For another experiment assessing synergy, an agar plate was utilized, which contained varying doses of antibiotics. Concisely, the experiment involved adding four antibiotics (fosfomycin, ciprofloxacin, oxacillin, and vancomycin) at low concentrations (0.25 μg/mL, 0.5 μg/mL, and 1 μg/mL) to the hard agar. Then, an overlay (DAOL) was conducted using soft agar harboring the phage vB_Sau_S90. The plates were incubated at 37°C for 16 h. The impact of antibiotics on the plaque morphology was noticed.

### Checkerboard analysis

The effect of the combination of phages and antibiotics was analyzed using the checkerboard method using phage vB_Sau_S90 and antibiotics: fosfomycin, ciprofloxacin, oxacillin, and vancomycin. Briefly, in a 96-well titer plate, phages from 10^2^ to 10^9^ PFU/mL were diluted along the abscissa and marked as plate A, and antibiotics from 64 to 0.125 μg/mL were diluted along the ordinates and marked as plate B. The phage dilutions from plate A were transferred to plate B in their respective wells (Hi-Media, India). To make the test bacterial isolate SA-90 inoculum, the overnight culture was diluted until it reached 0.5 McFarland turbidity. Then, 5 μL of the inoculum was added to each well that had the phage and antibiotic mixture. The effect of phage in combination with antibiotics was determined by a reduction in the optical density (OD_600nm_) as a stand-alone parameter, with the results converted to a percentage reduction value, and the results are represented as a heatmap. The effects of the combinations were calculated by measuring the fractional inhibitory concentration (FIC) index. The FIC index was calculated as described elsewhere (Manohar et al., 2022). The effect was interpreted based on the FIC index, which can be interpreted as synergistic (FIC <0.5), additive (2 > FIC ≤0.5), and antagonistic (FIC ≥2). The percentage of bacterial reduction at different combinations was calculated as follows: (OD of treated/OD of untreated) × 100%.

### Phage-antibiotic sequential treatment

By measuring the drop in bacterial turbidity, the study looked into how phages and sub-inhibitory levels of antibiotics work together. The combinatorial effect was evaluated in three different treatment sequences: a) administering phages before antibiotics (PRE), b) administering phages and antibiotics simultaneously (SIM), and c) administering antibiotics before phages (POS). For this study, SA-90 and phage vB_Sau_S90 were used. Briefly, the bacterial inoculum of OD_600nm_–0.2 (1 × 10^8^ CFU/mL), the phage at 10^7^ PFU/mL, and the antibiotics at a concentration of 1 μg/mL were incubated together in the aforementioned treatment sequences. The treatment sequence was maintained by adding phage at 0 min and antibiotic at 60 min in PRE-treatment, both phage and antibiotic at the same time (0 min) in SIM-treatment, and antibiotic at 0 min and phage at 60 min in POS-treatment. The reduction in bacterial turbidity was measured at OD_600nm_ every 2 h interval for 22 h. The effective treatment sequence was determined by calculating the fold reduction in bacterial growth, as described previously (Al-Anany et al., 2021). To compare the different treatment sequences (phage-antibiotic synergy, phage-alone, and antibiotic-alone), the percentage decrease in bacterial growth was found. Percentage reduction was calculated as follows: (OD of untreated—OD of treatment/OD of untreated) × 100.

### *In vivo* efficacy in *Galleria mellonella*

Study setup and treatment strategies: For *in vivo* studies, three bacterial isolates, SA-28 (strong biofilm producer), SA-90 (moderate biofilm producer), and SA-165 (weak biofilm producer), were used to assess the phage efficiency. The biofilm classification is based on weak biofilm production; ODc < OD ≤ 2ODc, moderate biofilm production; 2ODc < OD ≤ 4ODc and strong biofilm production; 4ODc < OD. Accordingly, two phages, vB_Sau_S90 and vB_Sau_S165, were used in combination with oxacillin. The efficacy of phage and antibiotic combinations in eliminating bacterial biofilms was evaluated. The efficacy of various antibiotics in combination with phages was evaluated *in vitro*, and oxacillin was found to be the most effective treatment. We tested how well the phage-oxacillin combination killed bacteria in three different treatment sequences: a) giving phages before antibiotics (PRE), b) giving phages and antibiotics at the same time (SIM), and c) giving antibiotics before phages (POS). Each treatment was performed independently with phage in combination with oxacillin. The concentrations used for the study were 10^9^ PFU/mL of phage in combination with oxacillin at 100 mg/kg. The experimental setup used for *G. mellonella* is shown in Supplementary Figure S1.

The efficacy of phage and oxacillin combinations in eradicating biofilms was evaluated in *G. mellonella* by introducing tooth bristles coated with biofilms. The study picked mature larvae that were creamy white in colour, measuring 20 mm in length, and weighing between 300 and 500 mg. Every study group comprised 10 larvae that underwent a 24-h period of starvation before the trial. In summary, to initiate biofilm development, the overnight culture was diluted at 1:10 with TSB supplemented with 1% glucose. This diluted culture was then introduced into 16-well titer plates that contained sterilized tooth bristles coated with 10% human plasma. The plates were subsequently incubated for 24 h. The tooth bristle was retrieved from the titer plate wells, cleansed (to eliminate free-floating cells), and aseptically introduced into the left pro-leg of the larva. The treatment was started 2 h after the injection, following the previously mentioned treatment sequence, i.e., PRE, SIM, and POS. In the PRE-treatment, the infected larvae were initially treated with the phages at time 0 and after 90 min with oxacillin. For SIM-treatment, both phages and antibiotics were administered simultaneously. For POS-treatment, the infected larvae were initially treated with sub-inhibitory concentrations of oxacillin at time 0 and after 60 min with phages. The control groups consisted of untreated, phage-treated, and antibiotic-treated groups.

The health condition of the larvae was monitored at 24-h intervals during a period of 5 days. The treatment’s efficacy was assessed by evaluating the larvae’s health using four key health index activities: cocoon formation, melanization, activity, and survival. The health index score was determined through observational assessment, wherein deceased larvae were assigned a value of 0, while the production of cocoons was regarded as a sign of better health and given a score of 1. The efficacy of the treatment procedures and phage combinations was evaluated in relation to the control groups. The survival rate was determined for both the treated and untreated larvae. The health condition and the advancement of infection in the larvae are illustrated in Supplementary Figure S2.

### Statistical analysis

The data shown are the mean value ±SD (n = 3). The treatment sequences’ outcomes were compared to the antibiotics’ outcomes using one-way ANOVA. The statistical distinction between the treatment sequence of an antibiotic and the treatment with only phage was assessed using the Student’s t-test, whereas the treatment sequences among the antibiotics were compared using a one-sample *t*-test. Statistical differences were assessed using a significance level of *p* < 0.05. The survival graphs were generated using GraphPad Prism.

## Results

### Not all antibiotics had a similar effect on the phage plaque size

**FIGURE 1:**
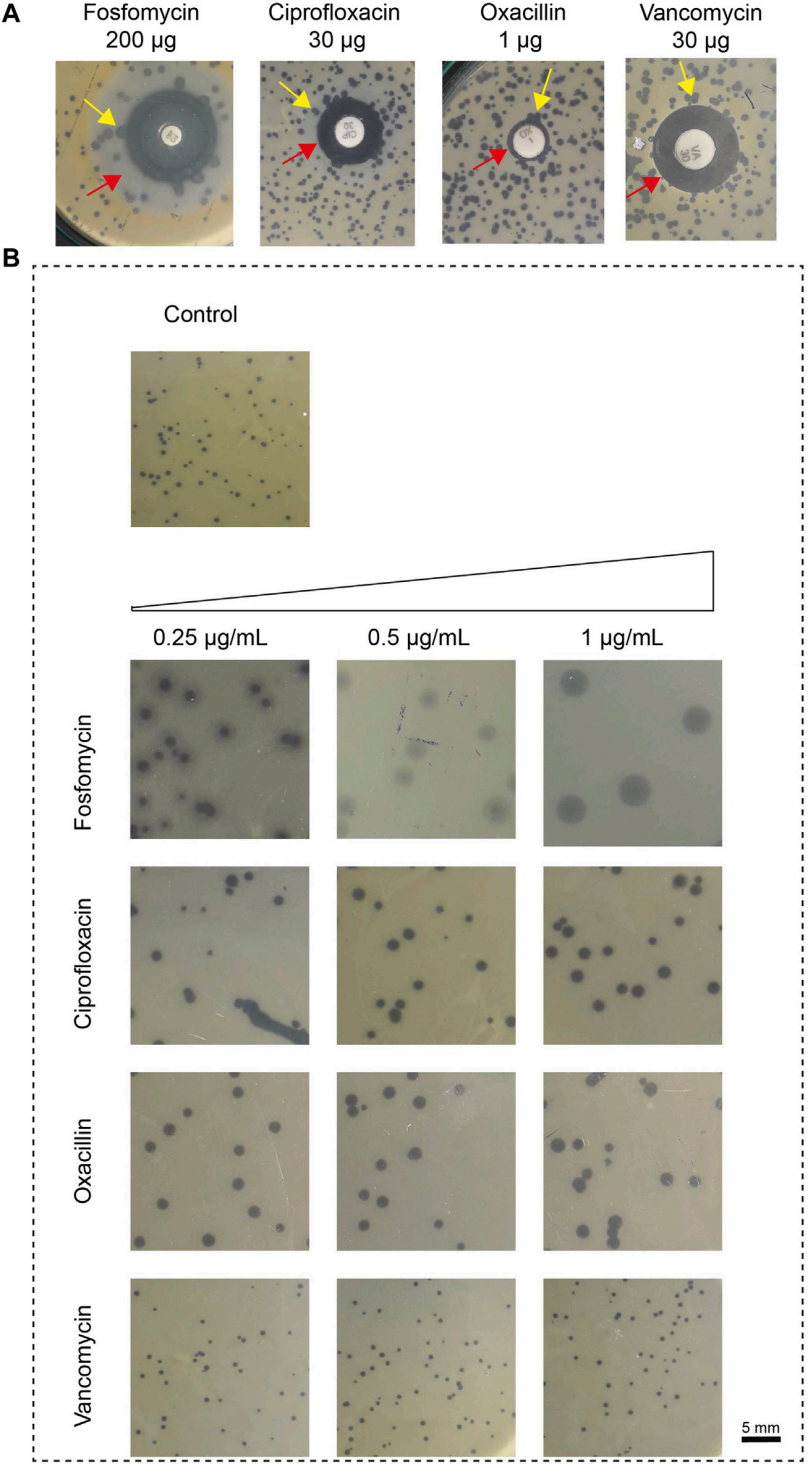
The impact of antibiotics, fosfomycin, ciprofloxacin, oxacillin, and vancomycin on plaque size. **(A)** Results of *in vitro* disk-diffusion synergy, the red arrow represents the sub-inhibitory concentration zone and the yellow arrow represents the variation in plaque size at the zone of sub-inhibitory antibiotic concentration; **(B)** Results of agar plate synergy at antibiotic concentrations, 0.25–1 μg/mL. The plaque size in the control group that had no treatment showed a plaque size of 0.5 ± 0.1 mm (top). Plaque size increased in the presence of antibiotic at 1 μg/mL; fosfomycin: 4 ± 0.5 mm, ciprofloxacin: 1.6 ± 0.1 mm, oxacillin: 1.6 ± 0.4 mm and vancomycin: no effect. The sub-inhibitory concentration was selected based on the Minimum Inhibitory Concentration (MIC), fosfomycin 8 μg/mL, ciprofloxacin 16 μg/mL, oxacillin 8 μg/mL and vancomycin 2 μg/mL.

We first determined the change in plaque size using various phage-antibiotic combinations in the antibiotic disc diffusion study. Here, we observed a change in plaque size near the subinhibitory antibiotic zone. The disc diffusion method was used as a preliminary test to confirm its effect on plaque size. The impact on plaque size was clearly observed near the subinhibitory zone. The plaque size variation with the antibiotic in the disc synergy method is represented in Figure 1A.

We next confirmed this effect by amending the sub-inhibitory concentrations of antibiotics (0.25 μg/mL, 0.5 μg/mL, and 1 μg/mL) on the DAOL plate. Here, the sub-inhibitory concentration was selected based on the Minimum Inhibitory Concentration (MIC), fosfomycin 8 μg/mL, ciprofloxacin 16 μg/mL, oxacillin 8 μg/mL and vancomycin 2 μg/mL. The sub-inhibitory concentrations analyzed were 0.25 μg/mL, 0.5 μg/mL, and 1 μg/mL and remained constant for all antibiotics studied. The plaque size in the agar plate synergy test is shown in Figure 1B. The antibiotic in combination with phages showed greater variation in plaque size for Fosfomycin (4 ± 0 mm). The plaque size in the control group that had no treatment showed a plaque size of 0.5 ± 0.1 mm. Plaque size increased gradually with increasing antibiotic concentrations. For fosfomycin, a maximum plaque size of 4 ± 0.5 mm was observed at 1 μg/mL. Other antibiotics had a minimal effect on the plaque size, showing 1.6 ± 0.1 mm and 1.6 ± 0.4 mm at 1 μg/mL with ciprofloxacin and oxacillin, respectively. No significant changes were observed with vancomycin at any of the studied concentrations (Table 1).

**TABLE 1:** The variation in plaque size at the different concentrations of antibiotics (0.25–1 μg/mL). The antibiotics were used at the sub-inhibitory concentrations and the represented data are the mean ± SD (n = 3) values of three independent experiments.

Antibiotic concentration	0.25 μg/mL (mm)	0.5 μg/mL (mm)	1 μg/mL (mm)
**Fosfomycin**	2.6 ± 0.4	3.5 ± 0.5	4 ± 0.2
**Ciprofloxacin**	1.2 ± 0.2	1.3 ± 0.1	1.6 ± 0.1
**Oxacillin**	1.1 ± 0.2	1.6 ± 0.4	1.6 ± 0.4
**Vancomycin**	0.5 ± 0.1	0.5 ± 0.1	0.5 ± 0.1

Minimum Inhibitory Concentration (MIC) of fosfomycin = 8 μg/mL, ciprofloxacin = 16 μg/mL, oxacillin = 8 μg/mL and vancomycin = 2 μg/mL.

### Phage-antibiotic combination is better than phages and antibiotics alone

**FIGURE 2:**
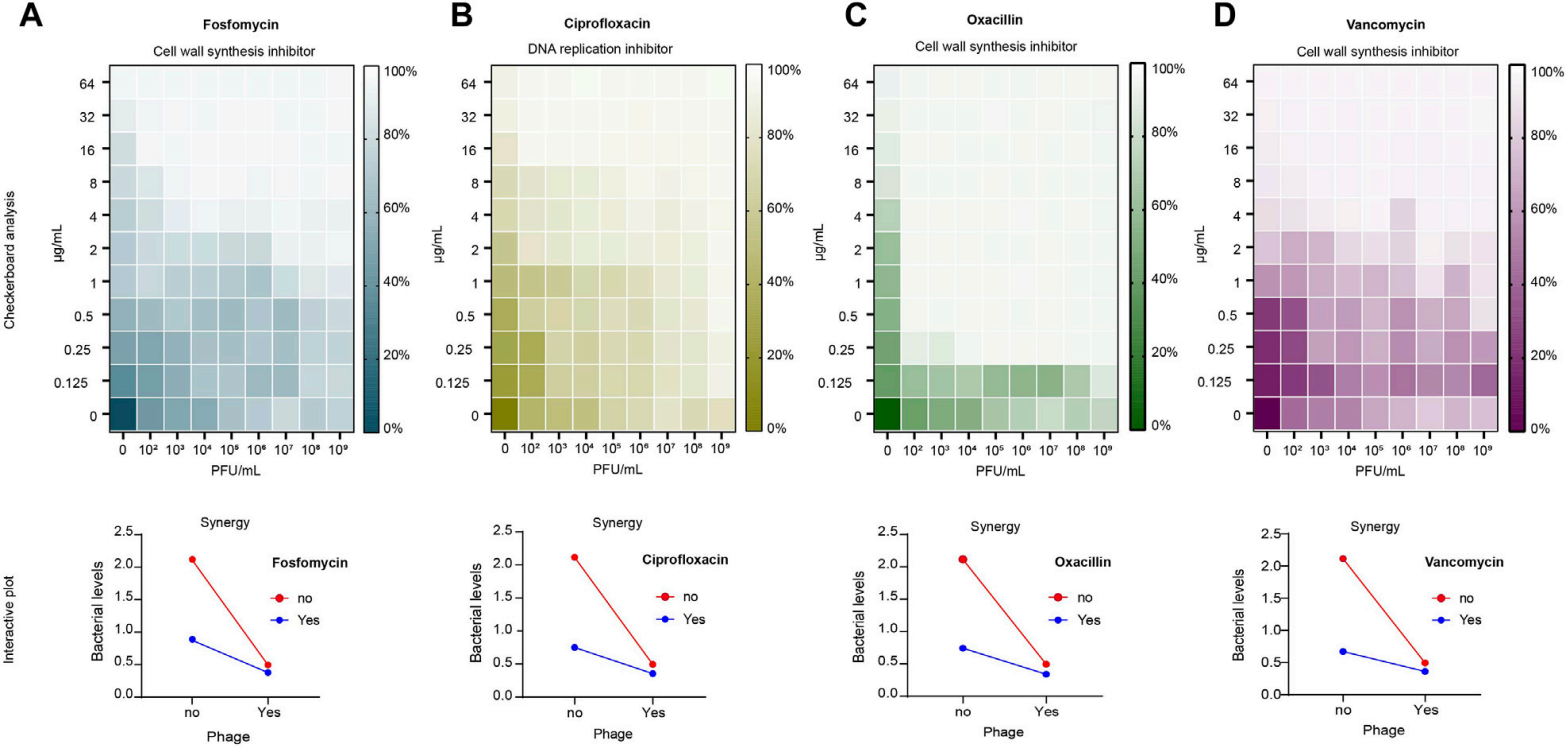
Checkerboard analysis of antibiotics and phage vB_Sau_S90. Antibiotic concentrations at the ordinate ranging from 0.125 to 64 μg/mL and phage concentrations at the abscissa (endpoint n = 16). The growth inhibition is represented as a heatmap: **(A)** fosfomycin; **(B)** ciprofloxacin; **(C)** oxacillin; **(D)** vancomycin. Interactive plot of the checkerboard analysis: **(A)** fosfomycin; **(B)** ciprofloxacin; **(C)** oxacillin; **(D)** vancomycin. Minimum Inhibitory Concentration (MIC) of fosfomycin = 8 μg/mL, ciprofloxacin = 16 μg/mL, oxacillin = 8 μg/mL and vancomycin = 2 μg/mL.

The results of the checkerboard analysis showed that the phage and all four antibiotics in combination had a synergistic effect (FIC index <0.5). The FIC indices of the antibiotics in combination with phage vB_Sau_S90 were 0.126, 0.072, 0.031, and 0.125 for fosfomycin, ciprofloxacin, oxacillin, and vancomycin, respectively. The combination of phages and antibiotics reduced the MIC by multiple folds compared to phages or antibiotics alone. For fosfomycin, the concentration of 8 μg/mL in the individual treatments was reduced to 1 μg/mL in combination with the phage. Similarly, for the ciprofloxacin antibiotic, it was reduced from 16 to 1 μg/mL, for oxacillin from 8 μg/mL to 0.25 μg/mL, and for the vancomycin antibiotic, it was reduced from 2 μg/mL to 0.25 μg/mL. The combinatorial effect of phage and antibiotic at different concentrations in checkerboard analysis is represented as a heatmap in Figure 2.

To compare the effects of the combination and individual treatments, a time-kill analysis was performed. Here, we considered the percentage reduction of simultaneous treatment as a phage-antibiotic combinatorial effect because most of the studies tested PAS by adding phages and antibiotics at the same time. Phage-antibiotic combination treatment resulted in more than an 80% reduction in bacterial growth, whereas phage alone showed a 76.6% reduction. In antibiotic-treated cells, the reduction was 58.5%, 64.5%, 64.8%, and 68.2% for fosfomycin, ciprofloxacin, oxacillin, and vancomycin, respectively. Under combinatorial treatment, fosfomycin efficiency increased 2.3 × times compared to antibiotic treatment alone and 1.3 × times compared to phage treatment alone. The ciprofloxacin-phage combination efficiency increased 2.1 × times compared to antibiotic-alone and 1.38 × times greater than phage-alone treatment. The oxacillin-phage combination efficiency increased 2.1 × times compared to the antibiotic-alone treatment and 1.45 × times greater than that of the phage-alone treatment. The vancomycin-phage combination efficiency increased to 1.8 × compared to that of antibiotic-alone treatment and 1.36 × greater than that of phage-alone treatment. The outcome of the checkerboard analysis is shown as an interactive plot in Figure 2.

### Administering phages before antibiotics was effective

**FIGURE 3:**
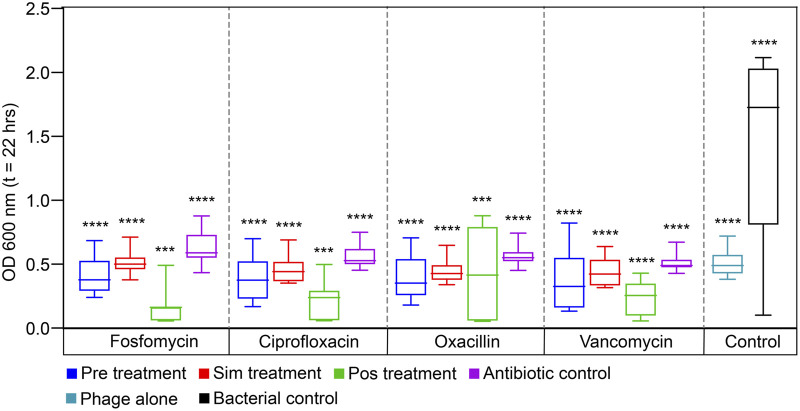
The reduction in the bacterial growth against three different treatment sequences of four antibiotics, fosfomycin, ciprofloxacin, oxacillin, and vancomycin are presented as a box plot (endpoint at OD_600nm_; t = 22 h). Pre-administering phages before antibiotics, Sim-administering phages and antibiotics simultaneously, Pos-administering antibiotics before phages, Antibiotic control-bacteria treated with antibiotic alone, Phage alone-bacteria treated with phage alone and Bacterial control-without treatment. Data are presented as mean ± SD (n = 3). A one-sample *t*-test was used for statistical analysis (treatment sequence among the antibiotics); *p* < 0.05, (^∗∗∗∗^p = <0.0001).

The results of the checkerboard analysis showed that the phage and all four antibiotics in combination were synergistic. The effectiveness of treatment decreased in the following sequence: POS > PRE > SIM. With fosfomycin, ciprofloxacin, oxacillin, and vancomycin antibiotics, respectively, the fold reduction for phage treatment after antibiotic treatment was 39.40, 37.05, 39.40, and 14.75-fold. Similarly, for the phage treatment followed by antibiotic, there were 8.93-, 7.47-, 9.19-, and 12.33-fold reductions with fosfomycin, ciprofloxacin, oxacillin, and vancomycin, respectively. The least reduction was seen in simultaneous treatment, ranging from 5.68-, 6.26-, 6.48-, and 6.27-fold with fosfomycin, ciprofloxacin, oxacillin, and vancomycin antibiotics, respectively. The statistical analysis showed that different treatment sequences among the antibiotics were significantly different, with a probability value less than 0.001 (statistical difference, *p* < 0.05). The fold reduction in bacterial growth among the antibiotics and treatment sequence is represented as a box plot in Figure 3.

The mechanism of action showed that the average fold reduction (average of all three treatment sequences) was high for fosfomycin and oxacillin, which stopped the production of cell walls (18.00 and 18.36 times, respectively), but low for ciprofloxacin, which stopped the production of DNA (16.93 times). However, the fold reduction of ciprofloxacin was found to be higher than that of vancomycin by 5.82-fold. The effect of PAS within the cell wall inhibitor showed that oxacillin resulted in the highest fold reduction of bacteria which was further chosen for *in vivo* studies.

### Phage efficacy against biofilms in *galleria mellonella*

**FIGURE 4:**
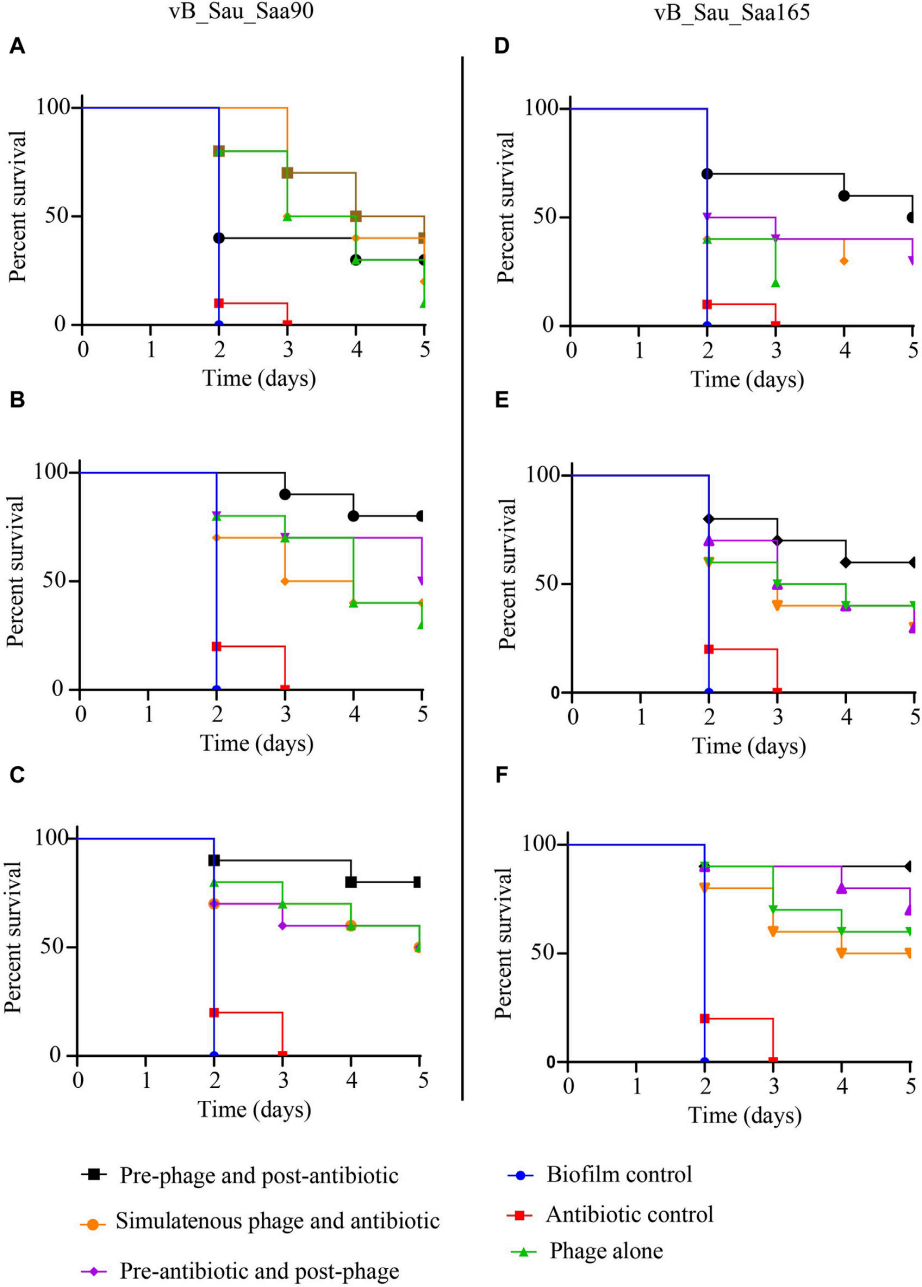
Impact of phage-antibiotic combinations on survivability of *Galleria mellonella* that was infected with *Staphylococcus aureus* biofilms and the effectiveness of different strategies of phage-antibiotic combinations, i.e., pre-phage and post-antibiotic, simultaneous phage and antibiotic, and pre-antibiotic and post-phage treatments. The treatment was initiated after 2 h of infection, whereas PRE-treatment = phages at time 0 and after 90 min with oxacillin, SIM-treatment = both phages and antibiotics were administered at time 0, and POS-treatment, oxacillin at time 0 and after 60 min with phages. For each treatment strategy, phages vB_Sau_S90 (A,B,C) and vB_Sau_S165 (D,E,F) were combined with oxacillin antibiotic at 100 mg/kg body weight.

The efficacy of two staph phages, vB_Sau_S90 and vB_Sau_S165, in combination with oxacillin was evaluated against biofilms in the *G. mellonella* model. Three different levels of biofilms (strong, moderate, and weak) assessed showed that all the infected larvae had 100% mortality within the third day (Figure 4). The larvae infected with SA-28, a strong biofilm producer, showed 10% and 20% survival with phages vB_Sau_S90 and vB_Sau_S165, respectively. The phage-oxacillin combination treatment showed higher efficiency when compared to the phage-only treatment. Among the three treatment strategies, administering phages before antibiotics (PRE) showed the highest survival percentage of 40% with vB_Sau_S90% and 50% with vB_Sau_S165. Followed by administering antibiotics before phages (POS) and administering phages and antibiotics simultaneously (SIM), showed closer outcomes ranging from 20% to 30% survival.

In the case of SA-90, a moderate biofilm producer, oxacillin showed 0% survival on day 3, while phage-only treatment showed 30% and 40% survival with phages vB_Sau_S90 and vB_Sau_S165, respectively. The phage-oxacillin combination treatment showed higher efficiency when compared to the phage-only treatment. Among the phage-antibiotic combination, administering phages before antibiotics (PRE) showed the highest survival percentages of 80% and 60% with vB_Sau_S90 and vB_Sau_S165, respectively. Followed by administering antibiotics before phages (POS) and administering phages and antibiotics simultaneously (SIM), showing closer outcomes ranging from 30% to 50% survival.

When the larvae were infected with SA-165, a weak biofilm producer, both the control and oxacillin-treated groups showed 0% survival on day 3, while phage-only treatment showed 50% and 60% survival with phages vB_Sau_S90 and vB_Sau_S165, respectively. Among the phage-oxacillin combination, administering phages before antibiotics (PRE) showed the highest survival percentages of 80% and 90% with phages vB_Sau_S90 and vB_Sau_S165, respectively. Followed by administering antibiotics before phages (POS) and administering phages and antibiotics simultaneously (SIM), showing closer outcomes ranging from 50% to 70% survival.

## Discussion

The findings demonstrate that the combined use of phages and antibiotics is more effective in eradicating bacterial load compared to using phages or antibiotics alone. The key finding of our study is that the combination of phage and antibiotics has a significant effect against *S. aureus*. The results of our study indicate that the variation in plaque size when antibiotics are added does not directly indicate synergism. However, the addition of certain antibiotics did lead to an increase in plaque size. Furthermore, not all antibiotics with the same mechanism of action produced similar results. Additionally, the sequence in which treatments were administered in the phage-antibiotic synergy (PAS) protocol was found to be a crucial factor in determining treatment success. Specifically, when phage treatment was followed by antibiotic treatment, it was more effective compared to other treatment sequences in PAS (Diallo and Dublanchet, 2022).

A key point to keep in mind about this study is that the addition of antibiotics made the plaques bigger. The effect on plaque size could be seen clearly in our disc synergy test, which was near the subinhibitory zone. This is based on the idea that the antibiotic from the disc slowly spreads into the nearby media, depending on how much antibiotic is in the disc. This makes a sub-inhibitory zone outside the inhibitory zone. The exact quantity at the sub-inhibitory zone, on the other hand, cannot be found. When different sub-inhibitory antibiotic concentrations were changed in the media, the biggest change in plaque size was seen at 1 μg/mL all four antibiotics that were tested. All of the antibiotics used in this study worked better when combined with phage vB_Sau_S90. However, the antibiotics that were used with the same phage all slowed bacterial growth by different amounts. We thought that these differences within the same phage might be caused by the way drugs work to help phage replication. We looked at the effects of phages, antibiotics alone, and the combination of the two to learn more about how they work together and what part they play in treating PAS. In our study, the plaque size increased significantly when phages were mixed with fosfomycin. However, there was no such big change seen when oxacillin and vancomycin were added, even though they work in the same way. The point of action of the same mechanistic groups can cause differences between them. When we look at how each antibiotic works exactly, fosfomycin acts in the initial step of cell wall peptidoglycan synthesis, inhibiting phosphoenolpyruvate synthetase (Petek et al., 2010). Oxacillin, on the other hand, inhibits binding to the PBP2a protein, which is the third or last stage of cell wall synthesis; vancomycin, on the other hand, stops cross-linking by binding to the D-Ala terminus (Hu et al., 2016; Goel et al., 2021). This shows that even within the same class of antibiotics, the point and time of action may be different. This can change how antibiotics work on the plaque.

When antibiotics were added to DAOL plates, the same effects were seen. We looked at the results of plaque size and the average fold decrease of bacterial growth during three different treatment sequences (PRE, SIM and POS) to see if increasing plaque size with antibiotics is a way to indirectly measure synergy. A high-fold reduction in bacterial growth was observed for oxacillin and fosfomycin, and the highest plaque size was observed only in the fosfomycin antibiotic disk. Although fosfomycin produced the largest plaque among the antibiotics studied, it showed the second-highest fold reduction in bacterial growth in the PAS test. Similarly, oxacillin produced a moderate change in plaque size and produced a high-fold bacterial reduction in the PAS test. In contrast, vancomycin did not have any effect on plaque size and similarly showed the least effect in combinatorial treatment. This suggests that plaque size production is independent and varies with the antibiotic; thus, it cannot determine the combinatorial effect in PAS analysis.

Preliminary and confirmatory tests were performed to determine the effect of antibiotics on plaque formation. These methods could have limitations for antibiotics with low diffusion ability. For instance, we hypothesized that the poor plaque size variation with vancomycin antibiotics could be due to their low diffusion. Similarly, when examining the effect of antibiotic susceptibility on the isolate, our study showed that the bacterial isolate SA-90 was susceptible to fosfomycin and vancomycin antibiotics. When comparing plaque size variation with the susceptible antibiotic, fosfomycin caused plaque size variation while vancomycin had no effect, suggesting that susceptible antibiotics cannot induce plaque size. A study conducted by Manohar *et al.*, showed that no plaque variation was observed in any of the antibiotic studies; however, the study showed that the reduction in bacterial burden with the same antibiotics in the time-kill analysis was significantly high (Manohar et al., 2022). In comparison with this study, it can be demonstrated that plaque size does not dictate synergism.

Recently, many studies have shown that phage-antibiotic combinations are more efficacious than phage-alone treatments (Huon et al., 2020; Van Nieuwenhuyse et al., 2022). Studies have shown that the synergism between phages and antibiotics varies broadly and could differ between the antibiotics and phages used (Comeau et al., 2007). To support our findings, previous studies showed that vancomycin does not work in the PAS test. For instance, a study by Dickey *et al.*, showed that vancomycin antibiotics antagonistically act in combination with *S. aureus* phages (Dickey and Perrot, 2019). Another study by Tkhilaishvili et al. showed no synergism with vancomycin (Tkhilaishvili et al., 2018). However, the efficacy of vancomycin was poor compared to other antibiotics used in this study, and the FIC index in the checkerboard analysis showed that vancomycin was synergistic. Similar to fosfomycin, oxacillin significantly reduces the bacterial burden. We found much of the study that supported our evidence of synergism between oxacillin antibiotic and *S. aureus* phages, a few examples being a study by Simon *et al.*, that showed the high efficiency of oxacillin and phage Sb-1 (Simon et al., 2021).

The sequence of treatment is known to affect the phage-antibiotic combination. Our data demonstrated that the treatment sequence had a large-fold variation in bacterial reduction. It was seen that planktonic cells were reduced more than 20 times when antibiotics were given before phage (POS). This observation is consistent with the findings of other studies (Kumaran et al., 2018; Dickey and Perrot, 2019). Although mechanistic interactions that enhance bacterial clearance in the presence of phages and antibiotics and their sequence of effects are unknown. Though it has been hypothesized that antibiotics can elongate bacterial cells, leading to increased phage production, the effect of the treatment sequence remains unclear (Liu et al., 2022). The effective antibiotics in the treatment sequence were fosfomycin > oxacillin > ciprofloxacin > vancomycin.

Biofilm-associated infections pose a significant challenge in clinical settings due to their inherent resistance to traditional antibiotic therapies. In this study, we examined biofilms of different strengths and assessed the survival rates of biofilm-treated larvae under various treatment conditions. We showed that the combination of phages with antibiotics exhibited superior efficacy compared to phage-only treatment. The presence of antibiotics in the treatment regimen likely contributes to enhanced bacterial killing by disrupting bacterial cell walls, rendering them more susceptible to phage infection. This synergistic effect was evident across all biofilm strengths evaluated in this study. Among the different combinations tested, the pre-phage and post-antibiotic treatment strategies consistently yielded the highest survival rates. This approach involves administering the phage prior to the antibiotic, allowing the phages to initiate biofilm degradation, followed by the antibiotic treatment to further suppress planktonic bacterial growth. This sequential administration may facilitate better biofilm penetration and improve bacterial killing efficacy. Similar findings have been reported in previous studies investigating phage-antibiotic combinations against biofilms (Lu and Collins, 2007; Ryan et al., 2011). Phage-antibiotic combination therapy is efficient in treating intricate infections caused by MRSA, including biofilm infections. Our research indicates that giving phages and antibiotics in a specific order with a minimum ∼3-h interval can improve the treatment’s efficacy.

## Conclusion

Our findings highlight the effectiveness of combining phages and antibiotics in treating *S. aureus*. The efficacy of treating planktonic cells using pre-antibiotic and post-phage methods was observed in laboratory conditions. However, when it comes to biofilms, the highest rates of larval survival were achieved with pre-phage followed by antibiotic treatment. The sequential administration of phages and antibiotics has been proven to be effective and warrants additional investigation for its efficacy against different infections. Additional research on the consecutive administration of phage-antibiotic can enhance our comprehension of the mechanisms behind these therapeutic interventions.

## Data Availability

The original contributions presented in the study are included in the article/Supplementary Material, further inquiries can be directed to the corresponding author.
