# Supplementary material for: Phage-antibiotic combinations in various treatment modalities to manage MRSA infections
Source: Front Pharmacol. 2024 Apr 9;15:1356179. doi: 10.3389/fphar.2024.1356179 (PMC11041375; doi:10.3389/fphar.2024.1356179)
Supplement: Supplementary file 1 [file DataSheet1.docx]

**Supplemental Figures:**


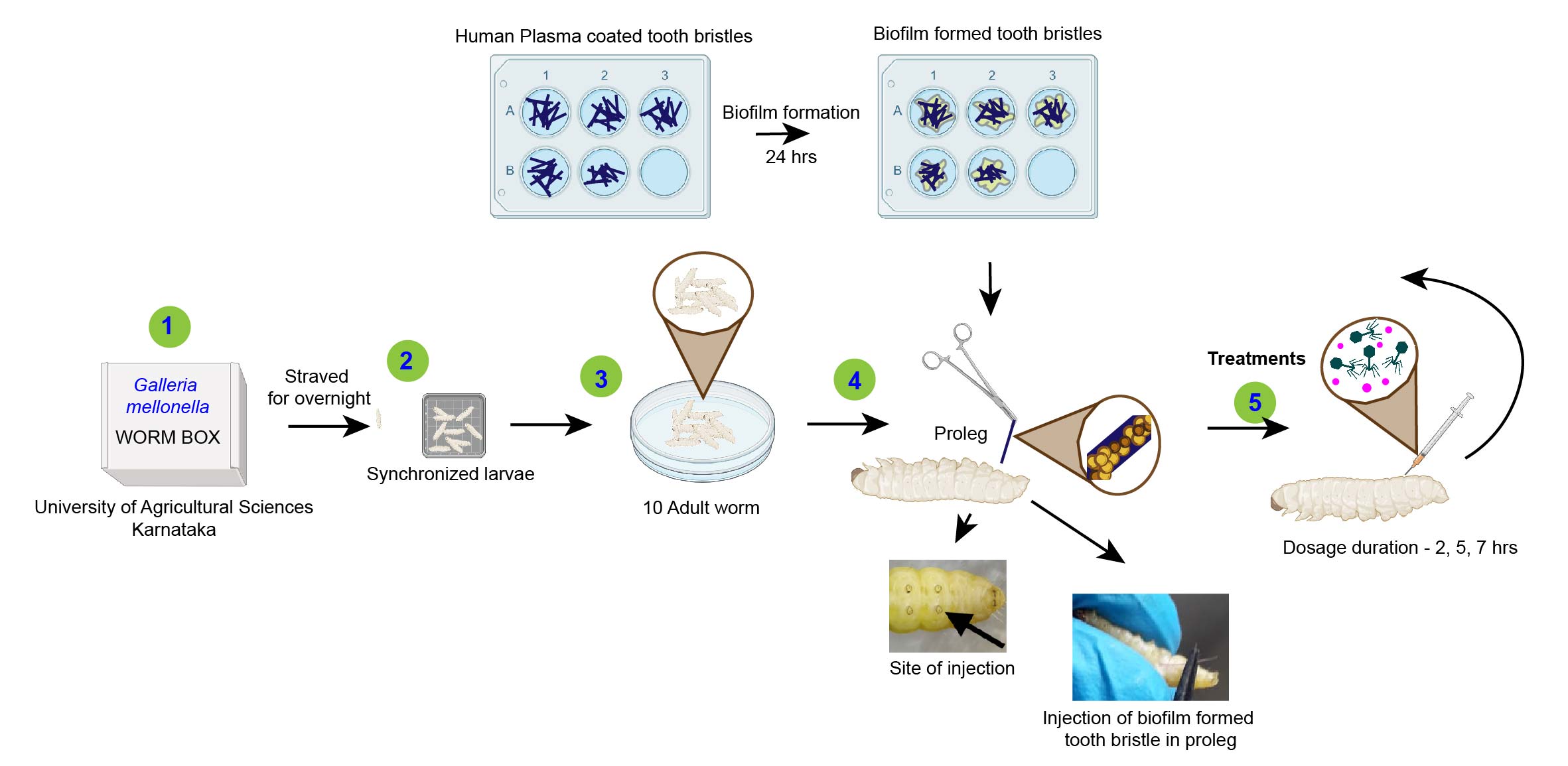


**Figure S1:** Schematic diagram of experimental setup used for *G. mellonella* biofilm model. 1. *G. mellonella* was harvested. 2. The adult (creamy white color) larvae were chosen and starved for 24 hrs. 3. The starved larvae were separated into different groups each consisting of 10 larvae. 4. The tooth bristle with biofilms are injected into the proleg of experimental larvae. Note: The biofilm coated tooth bristles should be prepared in advance. 5. The infected larvae were treated with phage-antibiotic combinations. In this study three distinct sequences were used i.e., pre-phage treatment, simultaneous treatment, and post-phage treatment at 90 min intervals.


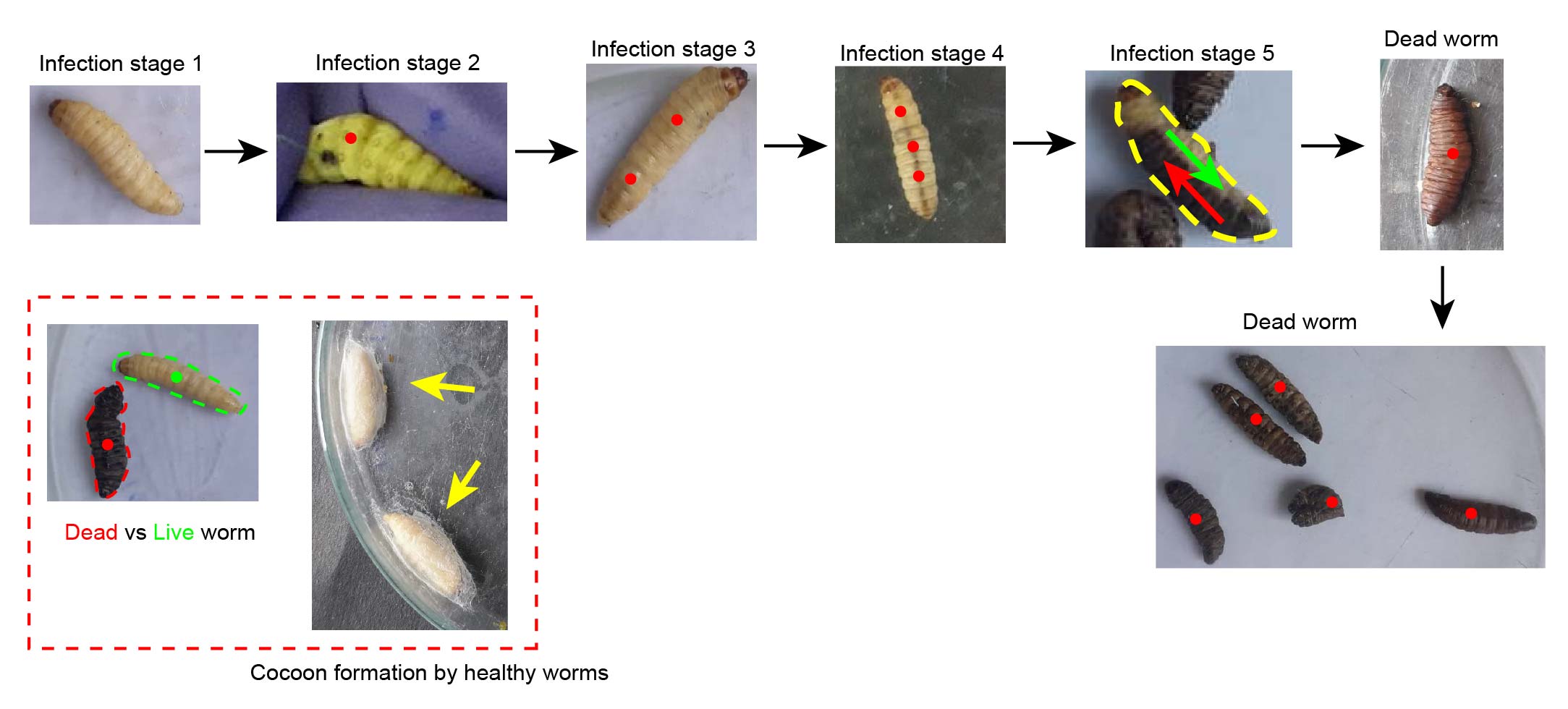


**Figure S2:** Different stages of infected *G. mellonella* larvae; live/ healthy larvae- creamy white colored and infected/ dead larvae- black spots/ black colored. Stage 1: Infected healthy larvae, Stage 2: Infected larvae with black spots, Stage 3 & 4: Infected larvae with more than one black spots, Stage 5: Infected dead larvae turning black. Cocoon-formation denotes the healthy larvae.
